# An Ultra-Fast Metabolite Prediction Algorithm

**DOI:** 10.1371/journal.pone.0039158

**Published:** 2012-06-20

**Authors:** Zheng Rong Yang, Murray Grant

**Affiliations:** Biosciences, College of Life and Environmental Science, University of Exeter, Exeter, United Kingdom; Université de Nantes, France

## Abstract

Small molecules are central to all biological processes and metabolomics becoming an increasingly important discovery tool. Robust, accurate and efficient experimental approaches are critical to supporting and validating predictions from post-genomic studies. To accurately predict metabolic changes and dynamics, experimental design requires multiple biological replicates and usually multiple treatments. Mass spectra from each run are processed and metabolite features are extracted. Because of machine resolution and variation in replicates, one metabolite may have different implementations (values) of retention time and mass in different spectra. A major impediment to effectively utilizing untargeted metabolomics data is ensuring accurate spectral alignment, enabling precise recognition of features (metabolites) across spectra. Existing alignment algorithms use either a global merge strategy or a local merge strategy. The former delivers an accurate alignment, but lacks efficiency. The latter is fast, but often inaccurate. Here we document a new algorithm employing a technique known as quicksort. The results on both simulated data and real data show that this algorithm provides a dramatic increase in alignment speed and also improves alignment accuracy.

## Introduction

Small molecules are the fundamental components of life, comprising the constituents of all biological material. Knowledge about the function, distribution and abundance of metabolites is fundamental to a comprehensive systems level understanding of an organism. Furthermore, soluble and volatile metabolites are central players in influencing interactions at a higher ecosystem level through their role in sensing, perception and elaborating biotic and abiotic stress responses. In post-genomic systems level research, the metabolome (all metabolites) of an organism is examined for various pattern analysis purposes [1] which will inform biological knowledge such as response to a particular stress or identification of molecular markers for medicinal or agricultural purposes. Multivariate analysis can be done using principal component analysis [2], [3], cluster analysis [4], [5], and discriminant analysis [6], [7] or for differential metabolite identification [7]. As a finger-printing technique, metabolomics can support the exploration of the relationship between metabolites and interactions influencing phenotypes, driving studies on metabolite network re-construction [8]. To ensure that these analyses are accurate and unbiased, it is necessary to make as precise a prediction of the mass and retention time of a unknown metabolite as possible. This is essential to *i*) the accuracy of compound recognition; *ii*) the accurate calculation of chemical composition of a metabolite [9]; and *iii*) the prediction of the function of unknown genes through metabolomics [8], [10], [11], [12], [13], [14].

Fundamental to any biological research, dynamic behaviour of biological molecules, be they proteins, mRNA or metabolites, needs to be determined through highly replicated experimentation. Metabolite features need to be first extracted from multiple mass spectra prior to any pattern analysis. Due to machine resolution and sample variation, one metabolite will have different implementations in different spectra, i.e. non-identical retention time and mass values. This means that the exact retention time and mass values of a real, but unknown metabolites may not be seen in collected spectra. Most metabolites are unknown therefore to accurately recognize metabolites, precise alignment of features across spectra is the first critical task in analyzing metabolomic datasets based upon accurate statistical estimations.

As described recently [15], three conditions must be satisfied for aligning features. First, features must fall within defined resolutions of retention time and mass to be considered for alignment. Second, no more than two features from the same spectra can be aligned to one consensus, i.e. the collision condition (Duran, 2003). The collision problem has been long been recognised and the resolution is normally equipment-dependent [16], [17], [18]. Third, mass shift cannot be ignored during alignment although we commonly ignore retention time shift, which is relatively small. All are critical to a reliable prediction (alignment) for multivariate analysis [15].

There are generally two types of alignment algorithms, i.e. a local merge strategy and a global merge strategy. The former commonly employs three techniques, warping [19], [20], [21], [22], [23], [24], nearest neighbour [25], [26] and clustering [19], [27], [28]. These are generally computationally efficient, but typically scan spectra one by one to generate consensuses, which cannot be updated or revised. Consequently the first scans may generate a false consensus based on an incorrect feature set which cannot subsequently be revised when “correct” features are scanned later [15], [28]. Many alignment tools, both commercial ones and freeware, belong to this type e.g. MetAlign [29], MSFACTs [26], OPenMS [30]. Binbase [31], MathDAMP [32], ChromA [24], LC-MSsim [33], XCMS [16], SpecAlign [34], MET-IDEA [35].

In order to increase alignment accuracy we recently developed PAD (Peak Alignment via Density maximisation), which adopted a global merge strategy [15] using a concept called the Map Coverage Maximization (MCM), where a `map’ refers to a spectrum. It implements a novel alignment principle, i.e. density maximisation. Among various overlapping candidate consensuses, a consensus with the highest density is selected as the prediction. A consensus refers to the prediction of a true, but unknown metabolite. However PAD is comparatively much slower than a local merge algorithm such as implemented by SIMA [28], which is typical to a global merge algorithm.

In this paper we present a novel feature alignment algorithm based upon the quicksort technique [36] used in computer sciences. The alignment run comprises four steps. The first converts features to a string list, which is then sorted. The second, similar to PAD, constructs candidate consensuses and detects their density. The third examines and filters the candidate consensuses to generate predictions. In the fourth step, features which fail to be aligned are put back to the string list and rerun. Here we evaluate this algorithm using both simulated data and real data. We conclude that this new algorithm is superior to currently available feature alignment algorithms in both alignment speed and alignment accuracy.

## Results

### Simulated Data – Toy A

Description of toy A is given in **Methods**. Table 1 shows the comparison of sensitivity (see **Methods** for the definition) analysis for Toy A data at noise levels 60%, 80% and 100%. For simulations with the noise levels below 60%, the sensitivity of all three algorithms is 100%. No data for specificity (see **Methods** for the definition) analysis is shown here because the specificity of all algorithms is 100%. From Table 1, we can see that at increasing noise levels (even within the allowed resolution), the sensitivity of SIMA consistently drops, from 97% to 72%, while both PAD and PASS maintains sensitivity at 100%.

**Table 1:** Sensitivity analysis of three sets (noise levels 60%, 80% and 100%) for Toy A.

60%			80%			100%		
SIMA	PAD	PASS	SIMA	PAD	PASS	SIMA	PAD	PASS
97.20%	100%	100%	86.14%	100%	100%	72.89%	100%	100%
96.17%	100%	100%	84.75%	100%	100%	73.61%	100%	100%
96.95%	100%	100%	86.70%	100%	100%	76.93%	100%	100%
96.61%	100%	100%	86.95%	100%	100%	73.98%	100%	100%
97.25%	100%	100%	84.75%	100%	100%	72.11%	100%	100%

### Simulated Data – Toy B

**Figure 1:**
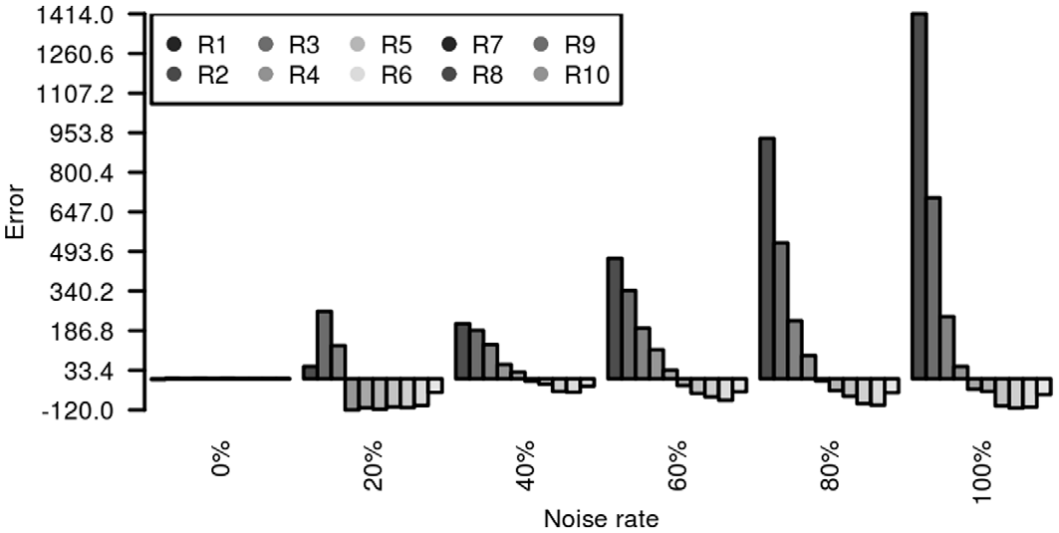
The distribution of prediction errors for Toy B data using SIMA (mass resolution 0.0071 Daltons). The horizontal axis represents the noise rate added to features in Toy B. The vertical axis represents either missing hypothesis (MH) or false prediction (FP). Each histogram group comprises ten bars representing ten types of consensuses (ten different number of features). The first bar represents the error between the number of expected singletons and the number of predicted singletons. The last bar represents the error between the number of true consensuses of size ten and the number of predicted consensuses of size ten. When FP occurs, we see a positive bar. When MH occurs, we observe a negative value.

Description of toy B is seen in **Methods**. No error (MH and FP - see **Methods** for the definitions) was observed for PAD and PASS for all six data sets. By contrast, when the noise level was increased from 0% to 100%, the prediction error in SIMA with the mass resolution (see **Methods** for the definition) 0.0071 Daltons got larger (Figure 1), leading to significantly increased singletons – see the trend of the first bars in Figure 1. Figure S1 shows the prediction error of SIMA with the mass resolution 0.00001 Daltons where we can see that the error is much more amplified.

### Real Data

A description of the real data is given in **Methods**. Table 2 shows the comparison of the CPU performance of the three algorithms using real data. CPU was measured in seconds. The first column indicates the alignments, for instance “Col.sid.6″ means aligning features of six maps for Col-0 and *sid2* at 6 hpi. The second column indicates the number of maps used for each alignment. The third column indicates the number of raw features in each alignment. The remaining three columns represent the CPU time in seconds for the three algorithms to complete the different alignments. The final column indicates the number of features reported in SIMA (mass resolution 0.0071 Daltons) outputs. The mass resolution used for running SIMA was 0.0071 Daltons. It can be seen that PASS is much faster than PAD (32 times faster) and also faster than SIMA (four times faster). It is important to note that features in original spectra files should not be duplicated nor omitted. PAD and PASS have generated alignments without these errors, however, SIMA generated alignments with duplicated and missing features. The last column of Table 2 contains the number of features reported in the SIMA output files. In theory, these numbers should concord with the numbers in column 3 of Table 2. However, 30% of raw features were missing when aligning the spectra of Col-0 and *sid2* at 6 hpi (hours post inoculation). Six duplicated features were found when aligning the spectra of Col-0 and *sid2* at 10 hpi. Six duplicated features were found when aligning the spectra of Col-0 and *sid2* at 16 hpi. Overall, 27% of features were missing when aligning 12 spectra of Col-0 at all three time points, 43% of features were missing when aligning 12 spectra of *sid2* at all three time points and the alignment of all 24 spectra delivered 17% duplicated features.

**Table 2:** Comparison of CPU times for the three algorithms to generate six alignments of the real data representing metabolite changes in *Arabidopsis thaliana* leaves infected with virulent *Pseudomonas syringae*.

Alignment	# maps	# features	SIMA CPU	PAD CPU	PASS CPU	SIMA prediction
Col.sid.6	6	54330	51	81	8	37767
Col.sid.10	6	56117	56	87	9	56123
Col.sid.16	6	66285	76	124	18	66291
Col	12	109369	244	1150	53	79301
Sid	12	119866	262	1544	57	68865
All	24	229235	1541	11598	360	267811

**Figure 2:**
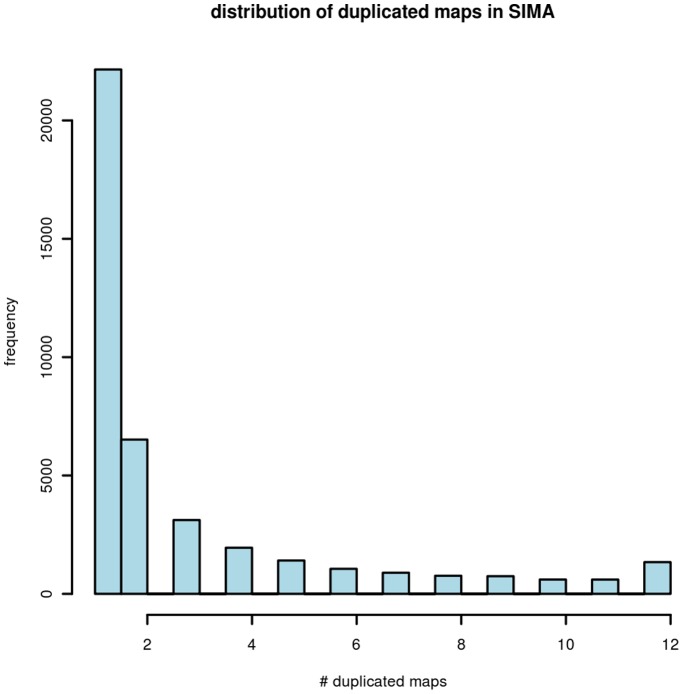
The distribution of duplicated maps in SIMA (mass resolution 0.0071 Daltons) consensuses alignment based on all 24 maps. The horizontal axis represents the number of Type-I errors in the generated consensuses. These range from one to 12. The vertical axis represents the frequency.

In addition, many SIMA consensuses violated the collision condition, i.e. many Type-I errors were found in SIMA alignments, e.g. containing more than one feature from the same map (spectra). Figure 2 shows the distribution of the number of duplicated maps in one consensus when aligning all 24 maps. It can be seen that the largest duplicated map number was 12, representing half of the total number of maps. Overall ∼10% of consensuses predicted by SIMA (mass resolution 0.0071 Daltons) contained duplications as denoted by the first bar in Figure 2. When using a mass resolution of 0.00001 Daltons for running SIMA, no such error was observed, but other types of error were amplified - see the discussion below.

**Figure 3:**
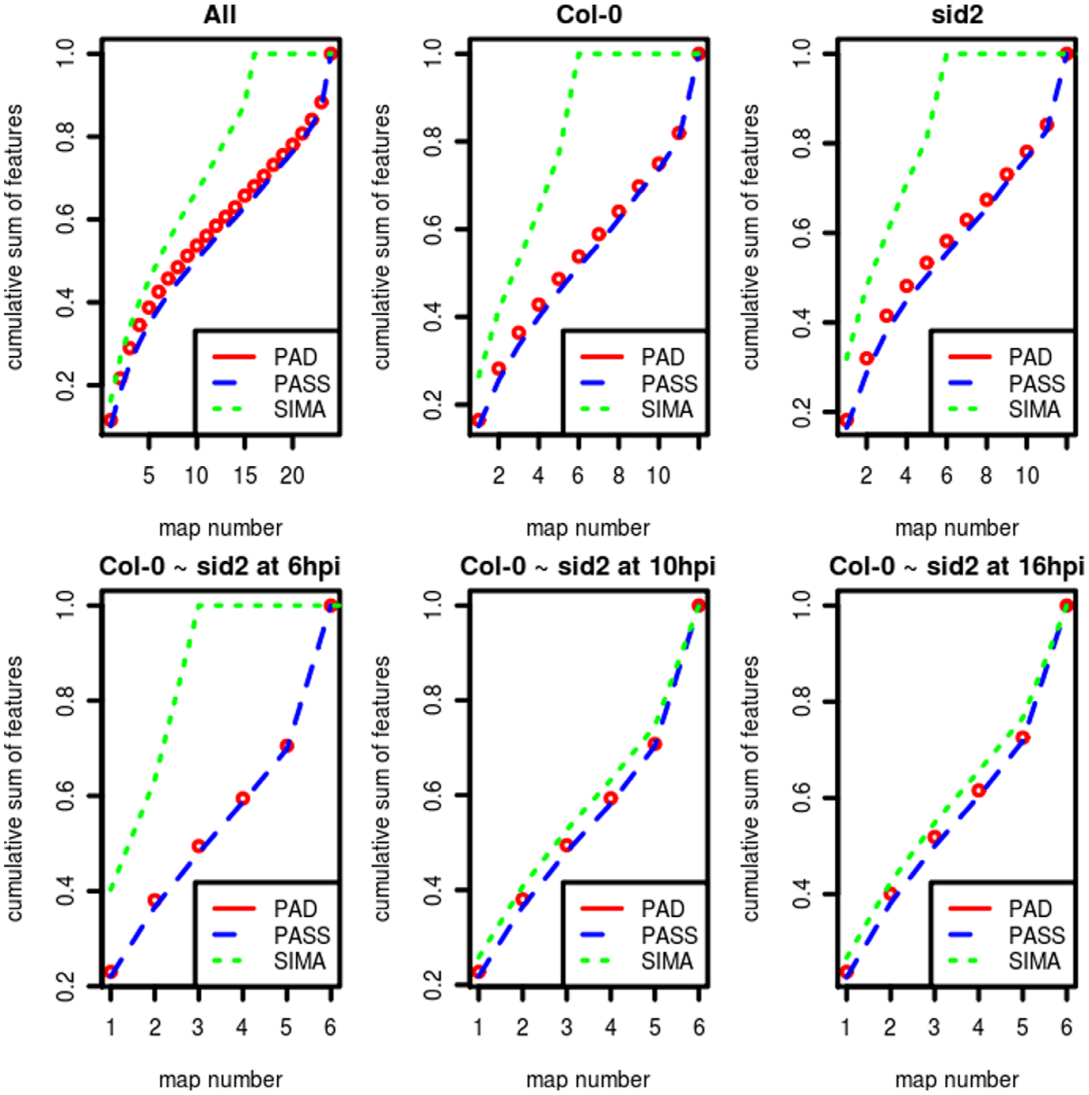
Characteristic alignment map (CAM) curves. The CAM was generated for MCM analysis of six alignments on the real data of pathogen infected plant leaves. The horizontal axes represent the maps used for each alignment, i.e. from six to 24. The vertical axes represent the cumulative sum of aligned features or the size of consensuses. The open dots represent CAM curves of PAD. Dashed lines represent CAM curves of PASS and dotted lines represent CAM curves of SIMA (mass resolution 0.0071).

As illustrated in Figure 3, the CAM (see **Methods** for the definition) curves of PASS are always the lowest and the CAM curves of SIMA (mass resolution 0.0071 Daltons) are always the highest. Notably four plots of SIMA show flat sections at the top, meaning that for these alignments, no large consensuses were generated, which was defined as the pattern IV (the biased H-pattern) in **Methods**. Figure S2 shows a comparison when running SIMA based on the mass resolution 0.00001 Daltons, where we can see that all CAM curves of SIMA are similar to the poorest performance, which was defined as the pattern I (the disastrous pattern) in **Methods**.

**Figure 4:**
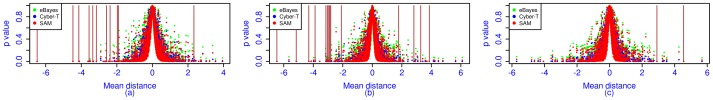
Significantly differential metabolites identified between Col-0 and *sid2* leaves responding to infection with *P. syringae* at 6 hpi, 10 hpi and 16 hpi. The horizontal axes represent the mean distance between Col-0 abundance and *sid2* abundance. The vertical axes represent *p* values. Each dot represents one metabolite. Each vertical line represents a significantly differential metabolite. (a) - top: Significantly differential metabolites between Col-0 and *sid2* at 6 hpi. (b) - middle: Significantly differential metabolites between Col-0 and *sid2* at 10 hpi. (c) - bottom: Significantly differential metabolites between Col-0 and *sid2* at 16 hpi.

The objective of improving alignment quality is to improve the quality of subsequent multivariate analysis. Accompanying this new alignment algorithm, we also introduce a novel significance analysis. Three widely used significance analysis algorithms; SAM [37], eBayes [38], and Cyber-T [39] were employed. The R program for detecting significantly differential metabolites is included in http://ecsb.ex.ac.uk/PASS. The prediction of significantly differential metabolites (between the *Arabidopsis* Col-0 wild type plant and salicylic acid deficient *sid2* mutant in this paper) was done via the consensus among the three algorithms. Figure 4 shows the distribution of significantly differential metabolites at 6 hpi, 10 hpi and 16 hpi. The use of this consensus approach can minimize the chance of a false prediction of differential metabolites because the three tests often disagree in terms of tail probabilities – small *p* values. Figure S3 illustrates such an example. With a simple consensus approach, we select predictions agreed by all three algorithms under a given significance level. In this study the significance level was set at 0.001 (this can be varied by the user when using our R code) leading to 11, 14 and 2 significantly differential metabolites for these three aligned data. They were shown as vertical lines in Figure 4. It should be noted that a metabolite with the largest mean differential abundance is not necessarily guaranteed to be predicted as being significantly differential. This is because the prediction does not only rely on the mean differential abundance, but also the variance. Here a differential abundance is the difference between the abundances of two treatments for a metabolite.

**Figure 5:**
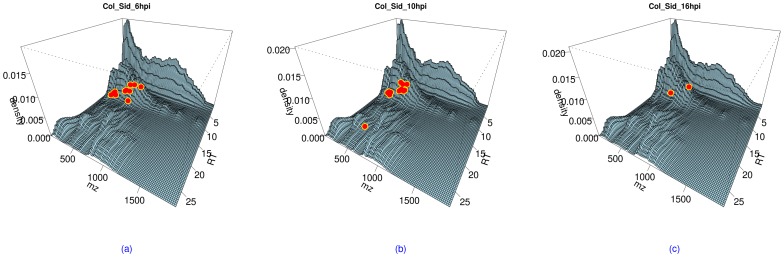
The location of significantly differential metabolites in R-M density surface. The significantly differential metabolites were shown using dots on the surfaces. (a) - left: for significantly differential metabolites between Col-0 and *sid2* at 6 hpi. (b) - middle: for significantly differential metabolites between Col-0 and *sid2* at 10 hpi. (c) - right: for significantly differential metabolites between Col-0 and *sid2* at 16 hpi.

The accompanying R program also supports locating significantly differential metabolites in a R-M (Retention time – Mass) density surface, i.e. where we can visualize the relationship between detected significantly differential metabolites and retention-time mass density. Figure 5 shows three plots for this visualization function.

Figure S4 illustrates the usage of the PASS program.

## Discussion

This paper has presented a new metabolite prediction (mass feature alignment) algorithm based on a widely used concept in computer sciences, the quicksort technique. The objective was to maintain the alignment accuracy based on the map coverage maximization principle, as recently described by Perera *et al.* in PAD (Perera et al. 2011), and to speed up alignment. PAD adopts a global merge strategy in contrast to many local merge algorithms, giving an improved alignment accuracy. Because a local merge algorithm has no regression process, its alignment is often problematic leading to poor alignment quality, which has two consequences, i.e. duplication and unreliable alignment. This was demonstrated here using SIMA, a typical local merge strategy algorithm. While a local merge algorithm is computationally fast, PAD, a typical global merge algorithm is not. We therefore implemented a quicksort approach, which is used in many programming languages, to speed up the global merge algorithm. Here we have built alternately M-clusters and R-clusters based on sorted mass and retention time values. Prior to building these two types of clusters, we converted all the numerical data including mass, retention time, metabolite abundance and spectra index to strings and organized them into a string list with recognizable labels to discriminate them. Applying the quicksort technique based on mass or retention time will not affect other domains of data and maintains a feature’s spectra index and abundance value during sorting. We additionally proposed a new technique for quantifying the quality of an alignment, i.e. Characteristic Alignment Map (CAM). Using CAM analysis, the alignment quality can be easily visualized qualitatively between different alignments. We have compared this new algorithm against PAD and SIMA using toy data sets and demonstrated that this new algorithm has improved alignment accuracy. Furthermore, we have shown using a real dataset that this algorithm has significantly improved alignment quality compared with SIMA and also has a better performance than PAD. Importantly, this new algorithm is 32 times faster than PAD and SIMA. The speed improvement has also been demonstrated theoretically in **Remark 3**. The most important concept for a global optimization process for peak alignment is consensus generation. Based on this study and our earlier work on PAD, it can be seen that a consensus must be a cluster of peaks with similar mass values and retention times which satisfy the resolution condition as well as the collision condition. Local optimization, as we have shown, will not be able to find all these peaks for one consensus. However comparing all peaks one by one is a typical NP (non-deterministic polynomial-time) - hard problem [43] as we saw in PAD. This is why the quicksort technique can significantly reduce the complexity leading to successful global optimization. Accompanying this alignment algorithm, we also introduced a novel approach for detecting significantly differential metabolites using a simple consensus principle to minimize the chance of delivering falsely predicted differential metabolites and visualizing the detected significantly differential metabolites.

## Methods

### Algorithm

The notations used by the algorithm are as follows: A data set is denoted by 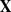, which is composed of *N* discrete features of *K* maps. Each map refers to a mass spectrum. Each feature 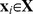 is a vector of four values, i.e. retention time 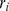, mass 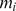, map index 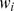 and feature intensity (abundance) 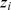. Retention time and mass reflect the chemical property of a metabolite and are used for predicting the chemical composition of a compound. The feature intensity is the reflection of the abundance of a metabolite and is the main parameter used in multivariate analysis, most notably differential metabolite predictions. The map index is only used to classify features, i.e. indicating from which spectrum a feature is collected. In addition to feature intensity, both 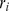 and 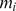 contain variation arising from both experimental and mass spectral resolution variation. The extent of variation is usually known.

It is also assumed that the observed features are random samples of a true, but unknown metabolite. This means that the following condition should be satisfied for an alignment of each feature
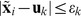
(1)where 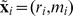 is the retention time - mass pair of a feature, which is an observed metabolite in a spectrum, 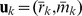 is the retention time - mass pair of a true metabolite, and 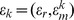 is the pre-defined resolution set (retention resolution and mass resolution). Here 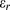 is commonly a constant (0.3 in this paper according to our mass spectrometer resolution) and 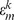 is variable, i.e. 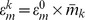. 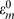 is a constant (10 ppm (its corresponding mass resolution is 0.00001 Daltons) in this paper as constrained by our mass spectrometer resolution), and 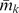 is the *k^th^* true mass under estimation. As each map may contain tens of thousands features, aligning features from many spectra becomes problematic in terms of speed - see Table 2.

Here we adopt a different strategy to speed up an alignment process dramatically while maintaining the alignment accuracy. In this algorithm, we still follow the resolution condition described in equation (1) and the collision condition. Following [15], we assume that the mass shift is linearly proportional to the true mass, i.e.
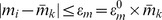
(2)


In theory, 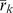 and 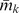 may not be exactly estimated. We therefore use their estimations, i.e. 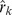 and 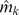, in an alignment process. A consensus is then expressed by (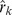, 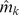).

The quicksort technique, a well known algorithm in computer sciences and implemented as a basic function in various programming languages, such as C, is used here to implement our algorithm. It sorts strings in a lexicographical order, i.e. the difference at an earlier position of strings has a priority compared with differences occurring at a latter position of strings. For instance, three strings AATT, ABAA and AAAA will be sorted to an order such as AAAA, AATT and ABAA. If strings represent numerical data, the order reflects the numeric accuracy of similarity, e.g. 130.034, 130.411, 130.410, 130.029, 130.411, 130.409, and 130.035 leads to 130.029, 130.034, 130.035, 130.409, 130.410, 130.411, and 130.411. At a mass resolution of 0.001, we can easily identify two clusters; (i) 130.033, 130.034, and 130.035 with the centre as 130.034 and (ii) 130.409, 130.411 and 130.410 with the centre as 130.410. The algorithm presented here was motivated by this observation. We note that this has been previously applied to proteomics studies [40], [41], where a single peptide mass was used for a targeted search within a data set of masses.

Mass spectral feature alignment is conducted in a two-dimensional space, reporting retention time and mass. We first designed a novel data structure to convert **X** to a string list **S** in which each feature is expressed using a string
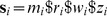
(3)where 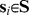. Using this notation, the dollar mark is used to separate four data domains. The use of the dollar mark will not affect a sorting process based on mass, which is at the first domain in the string list.

In order to guarantee an accurate sorting of data, all numerical data must be of the same length. If a feature's retention time (or mass) has lower than the maximal number of digits (decimals) then '0' is introduced to enable the sorting to function appropriately (e.g. 1.5 becomes 001.5000 if the maximum number of digits is three and the maximum number of decimals is four). We refer to such a numerical value (say 001.5000) as a digit-aligned-value (DAV).

**Figure 6:**
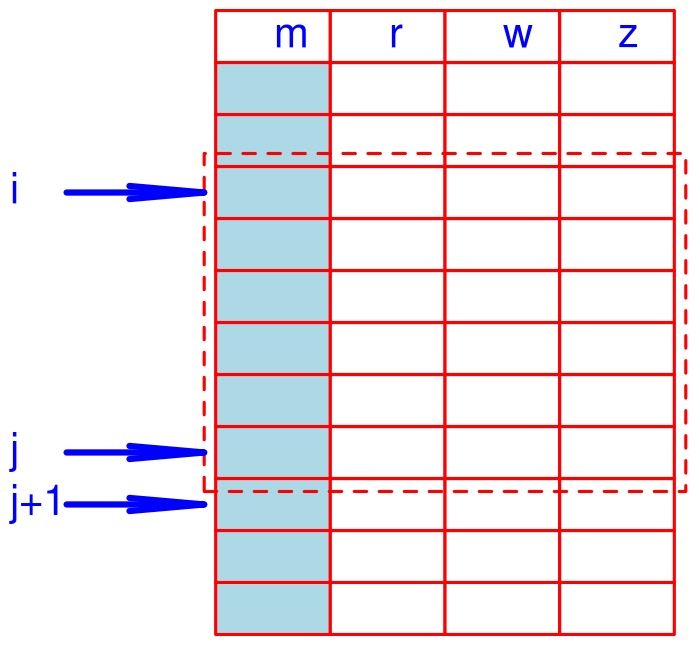
An illustration of constructing a M-cluster. The title line indicates the four fields of the string list; "*m*" stands for mass, "*r*" stands for retention time, "*w*" stands for map index, and "*z*" stands for feature intensity. The M-cluster starts from the *i* ^th^ string (row) and ends at the *j* ^th^ string (row). The dashed box indicates that the features (strings) within it form the M-cluster.

The alignment is run in two stages. In the first stage, we construct so-called mass clusters or M-clusters. Each M-cluster is composed of a number of features, which satisfy the enlarged mass resolution,
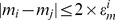
(4)where 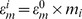. Figure 6 illustrates how a mass cluster is constructed, where retention time, map index and feature intensity are masked, hence not being used for the construction of this M-cluster.

**Figure 7:**
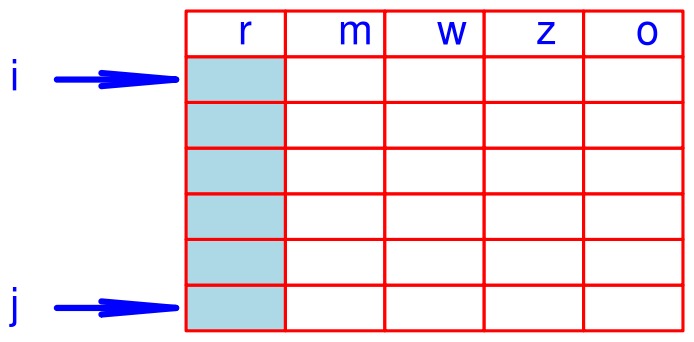
R-cluster formation in an 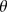-list. The first column stores retention time values of all features in the 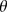-list. In addition to four columns, we have introduced the “o” column for indexing the **S** list.

An M-cluster is constructed by sequentially scanning the string list **S** till equation (4) is violated. For Figure 6, the scan was terminated or the M-cluster is constructed between *i* ^th^ feature and the *j* ^th^ feature if
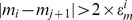
(5)The resolution is doubled in equation (5) because 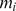 and 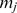 can be just on the two extreme boundaries of a consensus, i.e.
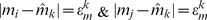
(6)where 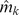 is the median mass of the kth consensus. Remark 1 below shows that this strategy is safe to construct an M-cluster as well as an R-cluster later. In addition, together with equation (7) given below, we call this strategy greedy scanning. Remark 2 below shows that this strategy almost guarantees the formation of an unbiased consensus. Staring from the j +1th string in S, the next M-cluster can be constructed. For each M-cluster, which is denoted by 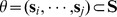, the second stage of this algorithm is to examine the retention time of the strings in 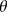 to construct retention time clusters or R-clusters. Note that there might be a number of R-clusters in one M-cluster because different consensuses may share very similar retention times as discussed in [15]. Prior to constructing R-clusters within one M-cluster, we have to move into another string structure to enable sorting retention time. In order to avoid any incorrect manipulation of the string list, we have to target this M-cluster locally. In practice, we simply copy the M-cluster to another string list shown in Figure 7, where we insert one more column (“o”) to remember where each feature (string) is copied from the S list. This reduced list is called a 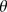-list.

**Figure 8:**
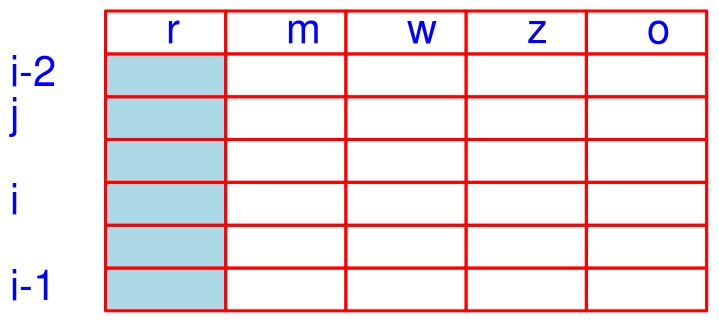
The 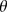-list after sorting based on retention time.

After sorting the retention time in the 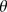-list, the original order of strings in the 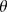-list will be changed. The use of the "o" column in this reordered 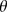-list (Figure 8) will save the information of the indexes to the **S** list, which is critical for later manipulations. As all the data including mass, map index, and feature intensity of a string (feature) are unchanged, these will shift concomitantly as string positions are resorted.

We next focus on forming R-clusters in the sorted 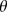-list. Starting from the first string in a sorted 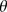-list, we scan features one by one to examine if the condition described below is satisfied
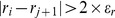
(7)We similarly double the retention time resolution as above because 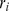 and 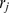 can reside on the two extreme boundaries of a consensus, i.e.
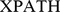
(8)where 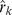 is the median retention time of the kth consensus. Staring from the j +1th string in a sorted 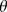-list, a next R-cluster will be considered. For each R-cluster denoted by 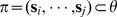, a consensus is constructed. For all features in 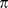, we calculate its median mass and median retention time using the following definition
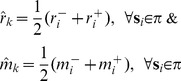
(9)where 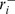 and 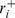 are the minimum and maximum retention times among all features in the current R-cluster (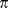). 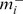 and 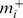 are the minimum and maximum masses among all features in 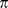. Deriving median mass and median retention time this way is designed to avoid possible bias [15].

**Figure 9:**
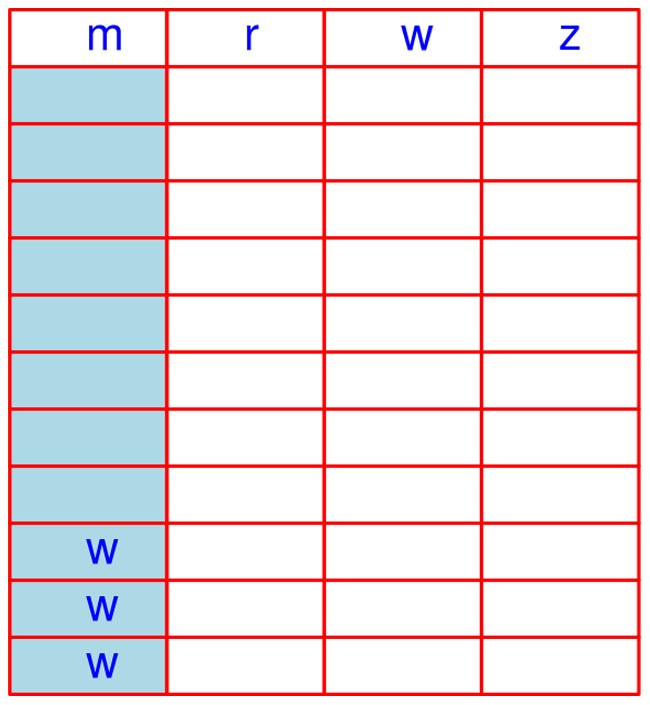
Example of “whitening” strings corresponding to aligned features. The rows with the “w” letter represent the strings of aligned features. Following quicksort these rows will be at the bottom of the **S** list and will not be re-visited in subsequent scans.

To save computing time, we always remove all the aligned features from the **S** list every time prior to running quicksort. To do so, we simply “whiten” all the strings corresponding to the aligned features by replacing the mass by the letter “w”. As the “o” column in the 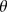-list records the original positive in the **S** list, it is very easy to trace them back to the **S** list to whiten the corresponding strings. After using the quicksort technique, all the strings of the aligned features (hence whitened ones) will be moved to the bottom of the **S** list automatically and will not be visited in subsequent scans (Figure 9).

When constructing a consensus, we need to mitigate two types of errors. A type-I error occurs when two features satisfy the resolution defined in equation (1) but are in the same spectra (map). A type-II error refers to the situation when a feature in a cluster does not satisfy the resolution defined in equation (1).

In order to follow the Map Coverage Maximization (MCM) principle [15], we first construct consensuses which cover all maps. When no further consensus can be constructed, we then look for consensuses, which cover *n* - 1 maps. This is repeated till one map is left. For instance, we will start finding consensuses of size ten if the total number of spectra is ten. If no consensus of size ten can be found, we search for consensuses of size nine, etc. In this way, we can ensure that the MCM principle is followed to generate reliable alignments.

The algorithm is implemented in C based on a linux computer with 3GB memory of 2.6 Ghz. The executable code is available at http://ecsb.ex.ac.uk/PASS.

### Remark 1


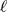
 DAVs in a sorted list corresponding to 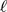 numerical values 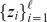 always follow a sequence of 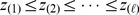, where 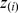 is the *i* ^th^ DAV in the sorted list.

**Proof:** We use the *reductio ad absurdum* approach for this proof. Suppose 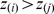, but 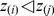. Here we use 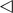 to denote an ascending order or lexicographical order, i.e. 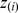 precedes to 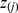 in a DAV list. For simplicity, we assume all values in a DAV list are integers. Generalizing the proof for values with decimals is straightforward. Suppose 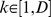 with *D* as the length of all DAVs is the first digit makes 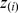 and 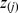 different. For instance, if two DAVs are 01312 and 01322, *k*  = 3 and *D*  = 4. We denote the two letters of these two DAVs at this position as 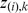 and 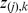. If 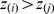, it is almost certain that 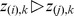. This means that 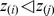 is not possible.

### Remark 2

The greedy scanning guarantees the formation of a consensus of all its features for a sorted list of mass and retention time values.

**Proof:** Again, we use the *reductio ad absurdum* approach for this proof. Suppose a feature list 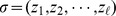 forms a consensus (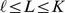 - *K* is the number of maps) and a sorted DAV list of it is expressed as 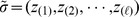, where 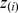 is the *i* ^th^ DAV in the sorted list. Based on the assumption that 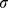 forms a consensus, 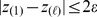 and 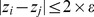, 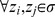, where 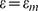 or 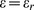. If one feature (denoted by 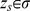) is beyond the cluster, it means that 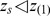 or 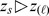. In other words, 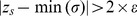 or 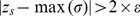. This is contrary to the assumption.

### Remark 3

The average time complexity of PASS follows 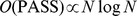 [42].

**Proof:** The time complexity of quicksort is 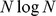. As it is difficult to estimate the metabolite distribution, we first assume that the features are equally distributed for consensuses of different size, i.e. the features are equally divided to form consensuses covering different numbers of maps. Importantly; *i*) we whiten corresponding strings in the **S** list whenever a consensus is formed; *ii*) quicksort is only used when the S list is exhausted. This means that the number of the strings in the **S** list when calling quicksort is decreased step by step as shown below (a note to the following equation is seen "**A notetoremark 3**" in the supplementary document)
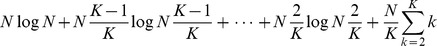
or



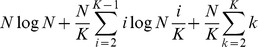
where the second component can be further re-written as



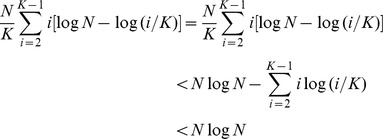
the last component of the above equation can be simplified as 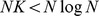 - see **remark S1** in the supplementary document. We next assume that all features contribute to singletons. In this case, only one quicksort is required and one scanning process of the **S** list is required. It is not difficult to see that the time complexity is 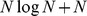. We finally assume that all features contribute to consensuses with full size, i.e. covering all maps. Following the **Remark** 2 discussed above, it can be seen that only one call to quicksort can guarantee the formation of all consensuses.

### Simulated Data Preparation

**Figure 10:**
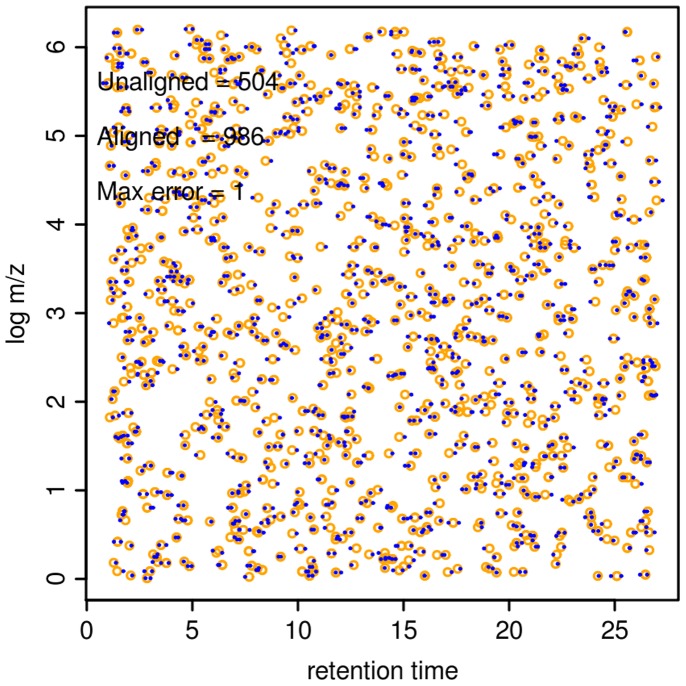
Distributions of features and TSMs in Toy A data. The circles represent TSMs and the dots represent the features in two maps. The two axes represent retention time and log*m/z* (or mass). The three lines of texts in the plots represent, in order; *a*) the number of features (non-aligned TSMs), which should not be aligned; *b*) the number of features (aligned TSMs), which should be aligned; *c*) maximum allowed noise level. A value of "1" means that noise was added to features at the maximum 100% of the pre-defined resolution, i.e. 0.3 min for retention time and 10 ppm for mass.

In addition to the simulated data used in PAD [15] (Toy B), an additional data set (Toy A) comprising two maps was used in this paper. In this new data set, “true simulated metabolites” (TSMs) were randomly generated using a retention time between 1 min and 27 min as well as mass between 1 and 500 following [15]. Two categories of TSMs were designed, i.e. non-aligned or aligned. Only two maps (spectra) were generated for analyzing both prediction sensitivity and specificity. The sensitivity is the percentage of aligned TSMs that are correctly aligned. The specificity is the percentage of non-aligned TSMs that are not aligned. For a non-aligned TSM, a feature was generated through adding random noise to both retention time and mass. These noise levels were sequentially 20%, 40%, 60%, 80% and 100% of the given resolution [15]. A feature of a non-aligned TSM was generated by
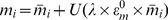
(10)and
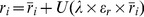
(11)where 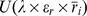 is the uniform distribution function with the interval defined as 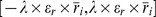, and 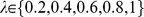 represents the noise level. The feature generated this way was then randomly distributed into one of two maps. For an aligned TSM, which in this case contains two features (because Toy A has two maps), each feature was generated through adding random noise and was distributed into one of two maps. Each feature was formed by both mass
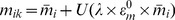
(12)and retention time
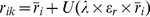
(13)where 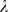 is as described above and 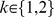. Figure 10 shows the distributions of features of one such data set, where 493 aligned TSMs (comprising 986 features) and 504 non-aligned TSMs were generated.

### Real Data Preparation

The data from [15] was used in this study for the comparison. The data is seen in ecsb.ex.ac.uk/PASS.

### Comparison of Algorithms

We used SIMA [28] and PAD [15] to evaluate the new algorithm as they represent the current benchmark for this type of application. Following [15], two mass resolutions (0.0071 Daltons and 0.00001 Daltons) were used to run SIMA for comparison one mass resolution (0.00001 Daltons) was used to run PAD and PASS. SIMA does not consider mass shift. We therefore follow PAD to use two mass resolutions for comparison.

**Figure 11:**
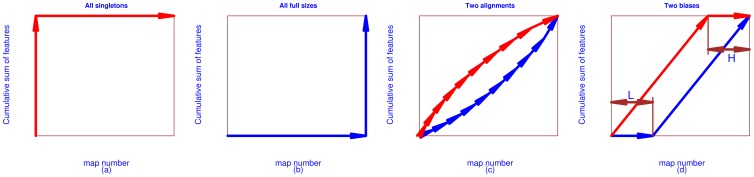
Two extreme and common examples of CAM curves. (a) Pattern I (disastrous pattern): all predicted consensuses are singletons; (b) Pattern II (perfect pattern): all predicted consensuses are of full size; (c) Pattern III (normal pattern): the comparison of two CAM curves for two alignments; (d) Pattern IV (biased pattern): two biased alignments. The upper one is defined as the biased H-pattern and the lower one is defined as the biased L-pattern. The horizontal axes represent the number of maps. The vertical axes represent the cumulative sum of features. (a) - panel 1: Pattern I; (b) - panel 2: Pattern II; (c) - panel 3: Pattern III; (d) - panel 4: Pattern IV.

### Sensitivity/specificity Analysis

To compare algorithms for these criteria we limited our analysis to Toy A data. We used the following assumptions. Suppose the number of non-aligned features is *N* and number of aligned features is 2*P*, *P* being the number of TSMs. If the observed number of singletons is *N*_0_ and the number of aligned consensuses is *C*_0_, then specificity is defined as
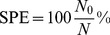
(14)and the sensitivity is defined as
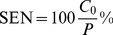
(15)


### Prediction Error – Missing Hypothesis (MH) and False Prediction (FP)

An alignment may introduce two prediction errors; a missing hypothesis (MH) or a false prediction (FP). A missing hypothesis means that a consensus of a specific size is lost during alignment (prediction). A false prediction means that an incorrect consensus is introduced for a specific consensus size. For simulated data (Toy B), we know in advance how many consensuses are expected. Post alignment, we have a set of consensuses, each formed by different numbers of features, corresponding to the consensus size. Suppose we have *K* maps, we use the following notation to denote the number of consensuses from 1 to *K* sizes, 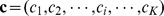, where 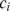 represents the number of consensuses of size *i*. In addition to the **c** vector, we define another vector of TSMs, 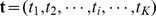, where 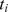 represents the number of TSM of size *i*. MHs occur when
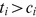
(16)and FPs occur when
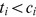
(17)Note that this measure only applies to a simulated data set where the t vector is known.

### Characteristic Alignment Map (CAM)

We introduced this for comparing algorithms on real data. Based on the **c** vector, we calculated the cumulative sum of features aligned to different consensus sizes. It was denoted by 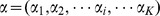 and 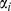 was defined by
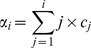
(18)


We used the map number as the horizontal axis and 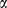 as the vertical axis to plot the data of 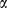. We referred to 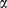 as the characteristic set and referred to this plot as a Characteristic Alignment Map (CAM) curve. In the worst case scenario, all predicted consensuses are singletons, i.e. being composed of a straight line in concord with the vertical axis first and a straight line in concord with the horizontal axis next – Figure 11 (a). This pattern is defined as Pattern I - disastrous pattern. A perfect alignment should generate CAM a curve touching the bottom-right corner, i.e. being composed of a straight line in concord with the horizontal axis first and a straight line in concord with the vertical axis next – Figure 11 (b). This pattern is defined as Pattern II - perfect pattern. Because many consensuses don’t occupy all maps, a CAM curve will stretch from the bottom-right corner towards to the top-left corner, i.e. between the two extreme curves - Figure 11 (c). This pattern is defined as Pattern III - normal pattern. In comparison, an alignment with a lower CAM curve is preferred compared with an alignment with a higher CAM curve, for instance the lower CAM curve in Figure 11 (c) is preferred. In Figure 11 (d), we show two biased alignments. They are defined as Patterns IV - biased patterns. The higher CAM curve shows the situation that the alignment losses consensuses with large sizes - H-pattern. If the map number is *M*, the alignment generates zero consensuses with sizes from *M* - *H* to *M*. The lower CAM curve illustrates that the alignment has no consensuses with small sizes - L-pattern. For map number *M*, the alignment generates zero consensuses with sizes from one to *L*. In theory, the total number of features before and after alignment should be identical. As SIMA was not reliable in this respect, the characteristic set (see **Methods** for details) was normalized for each algorithm in this paper for comparison, i.e.
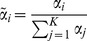
(19)where *K* refers to the number of maps (spectra). We then used 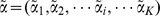 to investigate which alignment best follows the MCM rule [15].

## Supporting Information

Figure S1 **The distribution of prediction errors for Toy B data using SIMA (mass resolution 0.00001 Daltons).** The horizontal axis represents the noise rate added to features in Toy B. The vertical axis represents either missing hypothesis (MH) or a false prediction (FP). Each histogram group comprises ten bars representing ten types of consensuses, i.e. consensuses containing ten different features. The first bar represents the error between the number of expected singletons and the number of predicted singletons. The last bar represents the error between the number of true consensuses of size ten and the number of predicted consensuses of size ten. When FP occurs, we will see a positive bar (extending upwards from the horizontal axis). When MH occurs, we observe a negative value (extending downwards from the horizontal axis).
(TIFF)

Figure S2 **Characteristic alignment map (CAM) curves.** The CAM was done for MCM analysis of six alignments on the real data of pathogen infected plant leaves. The horizontal axes represent the maps used for each alignment, i.e. from six to 24. The vertical axes represent the cumulative sum of aligned features or the size of consensuses. The open dots represent CAM curves of PAD. Dashed lines represent CAM curves of PASS and dotted lines represent CAM curves of SIMA (mass resolution 0.00001 Daltons).
(TIFF)

Figure S3 ***p*** **value distributions of three modified** ***t*** **tests.** Both horizontal and vertical axes represent *p* values ranging from zero to one.
(TIFF)

Figure S4 **Instructions for using PASS.**
(TIF)

Remark S1 (DOC)
