# Supplementary figures and images for: An Ultra-Fast Metabolite Prediction Algorithm
Source: PLoS One. 2012 Jun 20;7(6):e39158. doi: 10.1371/journal.pone.0039158 (PMC3380062; doi:10.1371/journal.pone.0039158)

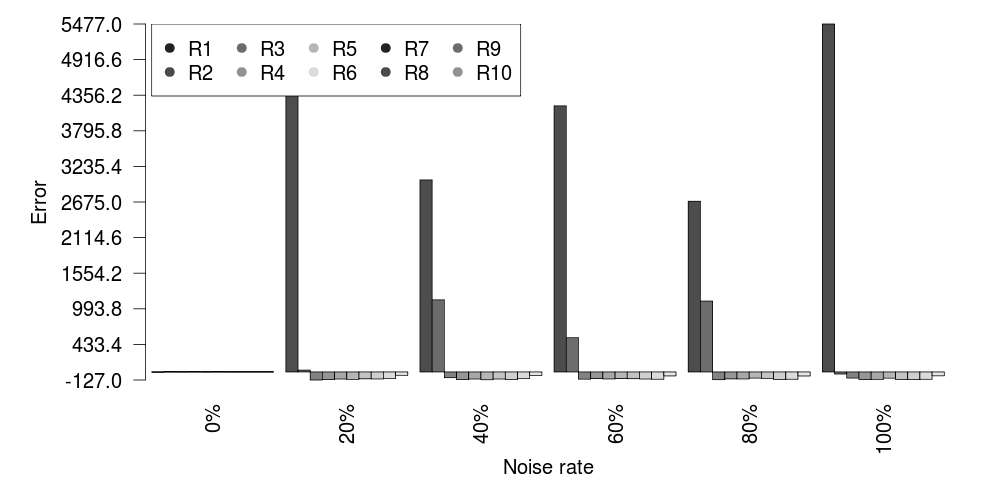

Supplement: Figure S1 — The distribution of prediction errors for Toy B data using SIMA (mass resolution 0.00001 Daltons). The horizontal axis represents the noise rate added to features in Toy B. The vertical axis represents either missing hypothesis (MH) or a false prediction (FP). Each histogram group comprises ten bars representing ten types of consensuses, i.e. consensuses containing ten different features. The first bar represents the error between the number of expected singletons and the number of predicted singletons. The last bar represents the error between the number of true consensuses of size ten and the number of predicted consensuses of size ten. When FP occurs, we will see a positive bar (extending upwards from the horizontal axis). When MH occurs, we observe a negative value (extending downwards from the horizontal axis). (TIFF) [file pone.0039158.s001.tiff]

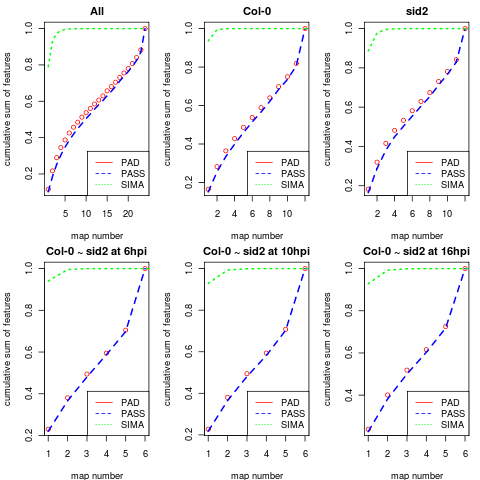

Supplement: Figure S2 — Characteristic alignment map (CAM) curves. The CAM was done for MCM analysis of six alignments on the real data of pathogen infected plant leaves. The horizontal axes represent the maps used for each alignment, i.e. from six to 24. The vertical axes represent the cumulative sum of aligned features or the size of consensuses. The open dots represent CAM curves of PAD. Dashed lines represent CAM curves of PASS and dotted lines represent CAM curves of SIMA (mass resolution 0.00001 Daltons). (TIFF) [file pone.0039158.s002.tiff]

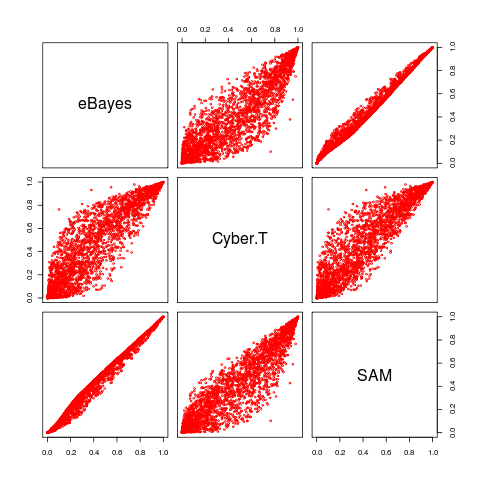

Supplement: Figure S3 — p value distributions of three modified t tests. Both horizontal and vertical axes represent p values ranging from zero to one. (TIFF) [file pone.0039158.s003.tiff]

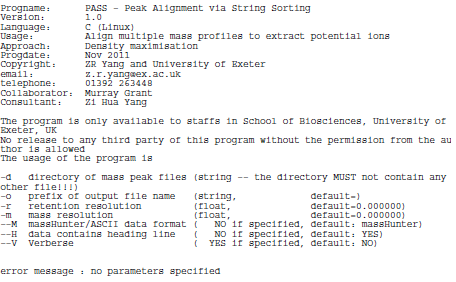

Supplement: Figure S4 — Instructions for using PASS. (TIF) [file pone.0039158.s004.tiff]
