# Supplementary material for: An Ultra-Fast Metabolite Prediction Algorithm
Source: PLoS One. 2012 Jun 20;7(6):e39158. doi: 10.1371/journal.pone.0039158 (PMC3380062; doi:10.1371/journal.pone.0039158)
Supplement: Remark S1 — (DOC) [file pone.0039158.s005.doc]

**Remark S1**

*Case I: equal distribution of features into consensuses*. Suppose the total number of features is *N* and the total number of maps is *K*. With the assumption of equal distribution and the use of MCM, we can see that there are *N* features in the first call to quicksort. Based on **Remark** 2, we can see that after the first call, $N/K$ features have been whitened because they have contributed to form $N/K^{2}$ consensuses of size *K*. After this first call to quicksort and whitening, $N-N/K=N(1-1/K)$ features are left in the **S** list. The second call to the quicksort will ensure $N-N/K-N/K=N(1-2/K)$ features to be used to form consensuses of size *K* - 1. Therefore, we have the following total time complexity to call quicksort under this assumption

$$N\log N+N\frac{K-1}{K}\log N\frac{K-1}{K}+\cdots+N\frac{2}{K}\log N\frac{2}{K}$$

In addition to the calls to quicksort, scanning the **S** list can be formulated as below. The first run scans *N* features, the second runs scans $N-N/K=N(1-1/K)$ features. Therefore the total number of scans is

$$N+N\frac{K-1}{K}+\cdots+N\frac{2}{K}=\frac{N}{K}\sum_{k=2}^{K} k=\frac{N}{K}\frac{(K-1)(K+2)}{2}\approx NK<N\log N$$
